# Risk factors of temperature increase after cytoreductive surgery combined with hyperthermic intraperitoneal chemotherapy

**DOI:** 10.3389/fonc.2023.1120499

**Published:** 2023-03-22

**Authors:** Hui-xia Kang, Jun-ying Ma, Yan-yan Su, Shan Kang, Bao-jie Feng, Xiao-bei Feng, Xu-sha Wang, Yun-yun Lu

**Affiliations:** Department of Gynecology, The Fourth Hospital of Hebei Medical University, Shijiazhuang, China

**Keywords:** CRS, HIPEC, temperature increase, risk factors, analysis

## Abstract

**Background:**

Cytoreductive surgery combined with hyperthermic intraperitoneal chemotherapy (CRS-HIPEC) is the standard treatment for patients with peritoneal cancer (PC). Following CRS-HIPEC, patients may also face risks caused by whole body hyperthermia. This study analyzed the incidence of temperature increases following CRS-HIPEC and identified the attendant risk factors.

**Methods:**

A retrospective analysis was carried out among 458 patients who received CRS-HIPEC at the Fourth Hospital of Hebei Medical University between August 2018 and January 2021. The patients were divided into two groups according to post-HIPEC axillary temperature (≥38°C), with the demographics and the laboratory test results subsequently analyzed and compared, and the risk factors pertaining to temperature increases analyzed using univariate and multivariate logistic regression.

**Results:**

During CRS-HIPEC, 32.5% (149/458) of the patients with a temperature increase had an axillary temperature of not lower than 38°C, and 8.5% (39/458) of the patients with hyperpyrexia had an axillary temperature of not lower than 39°C. Female gender, gynecological malignancies, type of chemotherapy drug, increased postoperative neutrophil percentage, and a sharp drop in postoperative prealbumin were associated with the incidence of a temperature increase and axillary temperatures of >38°C. Among these factors, the type of chemotherapy drug was identified as an independent risk factor for a temperature increase during CRS-HIPEC.

**Conclusion:**

By determining the risk factors pertaining to temperature increases during CRS-HIPEC, medical staff can identify the attendant risks among the patients and thus take preventive measures in a timely manner to maintain the patient’s body temperature at a stable level. This suggests that further clinical research should be conducted to build a risk-prediction model for temperature increases following CRS-HIPEC.

## 1 Introduction

Peritoneal cancer (PC) is a common clinical manifestation of advanced gastrointestinal and gynecological malignancies and peritoneal mesothelioma, one that seriously affects the long-term survival of patients. Relevant research has been carried out since the early 1990s to develop therapeutic procedures for peritoneal surface malignancies. Cytoreductive surgery (CRS), which was first defined by Sugarbaker (1), includes multiple organ resection and peritoneum resection. Here, the objective is to resect primary lesions, organs, and/or peritoneal surface metastases as completely as possible to ensure that there is no visible lesion remaining in the abdominal cavity. However, simply resecting all visible tumors may not be sufficient, as the remaining microscopic diseases can result in postoperative relapse. Post-operative intraperitoneal chemotherapy (IPC) is aimed at complete macro-to-micro CRS, and CRS combined with IPC has been recognized as the standard of care for patients with PC, including in terms of pseudomyxoma peritonei, appendiceal adenocarcinoma, gastric cancer, colorectal cancer, and peritoneal mesothelioma (2). This combination of CRS and IPC allows for the palliative treatment of peritoneal malignancies to become curative. There are two main methods of IPC, namely, early postoperative intraperitoneal chemotherapy (EPIC) and hyperthermic intraperitoneal chemotherapy (HIPEC). In EPIC, chemotherapy drugs are perfused into the abdominal cavity through a catheter placed on an abdominal wall proximate to a site with the greatest risk of recurrence within 1–5 days following CRS (3). In HIPEC, which is essentially IPC combined with hyperthermia, moderate hyperthermia of above 41°C can deliver a direct anti-tumor effect by increasing the cytotoxicity of certain chemotherapy drugs and the depth of penetration to tumor nodules *via* chemotherapy. As such, long-term survival has been achieved among patients treated with HIPEC following CRS (2).

Large-scale resection, physical or chemical trauma, and HIPEC change the capillary permeability, which leads to postoperative abdominal and systemic complications due to tissue injuries, increasing the incidence of postoperative complications and the risk of death, and prolonging the length of stay (LOS) and the postoperative recovery period. The incidence and mortality rates of these complications are 22%–41% and 2%–5%, respectively. The complications include superficial/deep wound infection, renal insufficiency, myocardial infarction, deep vein thrombosis, and sepsis (4, 5). Following CRS, HIPEC patients also face the risk of whole body hyperthermia. A higher core body temperature (CBT) is an independent risk factor for increased postoperative complications. The possibility of postoperative complications increases by 2.68 times with every 1°C-increase in CBT (6). During HIPEC, a temperature that increases to 38°C for 2 h can result in a higher level of metabolic activity, heart rate, and end-tidal carbon dioxide concentration, as well as metabolic acidosis, which ultimately leads to oxygen depletion (5).

The above indicates that maintaining a normal body temperature during HIPEC is a crucial goal of perioperative management. This study analyzes the incidence of temperature increases following CRS-HIPEC and identifies the attendant risk factors in view of providing a theoretical basis for clinical care.

## 2 Materials and methods

### 2.1 Demographics of patients

**Figure 1:**
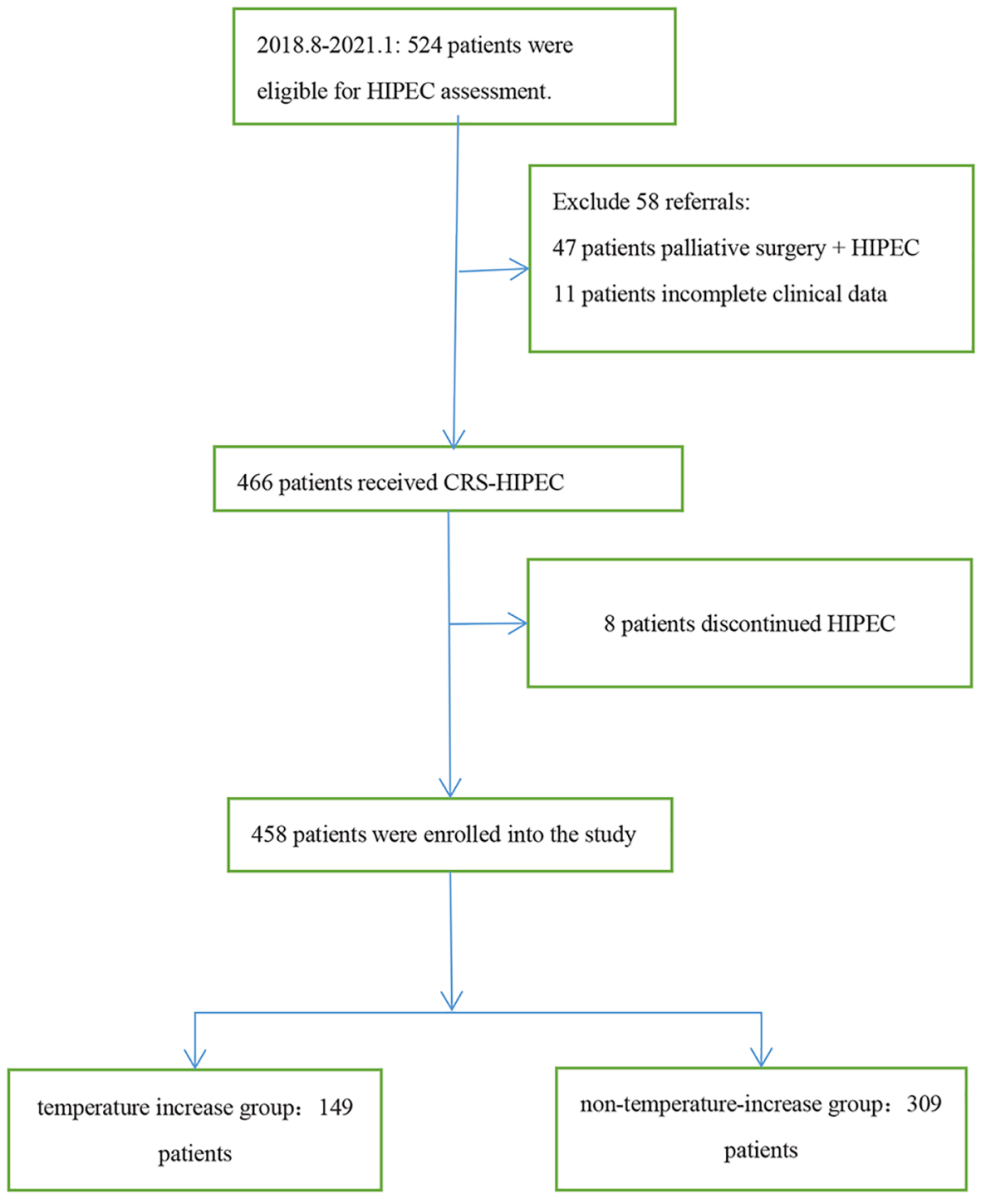
Flowchart of patient enrollment and study population.

This was a single center, retrospective study, involving 458 patients diagnosed with malignant tumors based on pathology who received 1–5 rounds of HIPEC following CRS in the Fourth Hospital of Hebei Medical University from August 2018 to January 2021. The indications of HIPEC included the following:curative intent of peritoneal metastases from primary or recurrent ovarian cancer, colorectal cancer, gastric cancer, peritoneal mesothelioma, pseudomyxoma peritonei and other malignancies with peritoneal metastases. All patients were informed of the risks and signed the informed consent form prior to HIPEC, and the study was approved by the ethics committee of the hospital. The inclusion criteria of patients were 18 years old and over, and all had CRS-HIPEC indications. Target populations were subjected to histodiagnosis based on the relevant guidelines (7), and patients who had received palliative surgery + HIPEC, those with incomplete clinical data, and those who discontinued HIPEC were excluded (**Figure 1**).

### 2.2 Cytoreductive surgery and hyperthermic intraperitoneal chemotherapy

All the patients underwent surgical treatment, including radical resection, CRS, and tumor resection. Before the abdomen was closed, four disposable body-cavity hyperthermic perfusion tube assemblies (Type D) (including two inflow tubes and two outflow tubes) were placed crosswise into the upper and lower quadrants of the abdominal cavity. The patients’ physical conditions were evaluated by medical professionals prior to HIPEC, with the operation performed by professionally trained nurses, typically 1–2 days after the surgery. The doctor in charge developed 1–5 HIPEC plans according to the intraoperative conditions, with intervals of a minimum of 24 h. The automatic hyperthermic perfusion equipment used was the body-cavity hyperthermic perfusion treatment system (model: BR-TRG-II), and a disposable body-cavity hyperthermic perfusion tube (model: BR-TRG-II), both of which were supplied by Guangzhou Baorui Medical Technology Co., Ltd. Sedative and analgesic drugs were given to the patients 30 min prior to the treatment to alleviate their tension and anxiety. A closed hyperthermic circuit system was established with two inflow tubes and two outflow tubes between the equipment and the abdomen, and 4,000 ml of glucose or normal saline solution maintained at 43°C ± 0.1°C was perfused into the abdomen at a circulating pump flow of 400–600 ml/min. The chemotherapy drugs to be perfused were first dissolved in the perfusate. Each HIPEC procedure lasted 60 min, and the catheters were removed one by one after the treatment.

### 2.3 Outcome indicator

The main outcome indicator of this study was the temperature increase of the patients within 0-120 min following HIPEC commencement, which was defined as an axillary temperature of not below 38°C. Moderate fever was defined as 38°C–38.9°C and high fever as not below 39°C. The axillary temperature was measured at (1) the beginning and (2) the end of the HIPEC, and (3) 30–60 min after the procedure.

### 2.4 Statistical analysis

The demographic characteristics of the patients, the histology classification, the disease characteristics, and the clinical laboratory results were summarized using descriptive statistics. The data were subjected to statistical processing using SPSS26.0 software, with the measurement data expressed as mean ± standard deviation or median. Inter-group comparisons were performed using an independent-samples *t*-test. The enumeration data were expressed in terms of case and percentage, with inter-group comparisons performed using a *χ* ^2^ test or Fisher’s exact test. Statistically significant variables in the univariate analysis were included in the logistic regression model for multivariate analysis, and the difference was statistically significant when *P* < 0.05.

## 3 Results

### 3.1 Patient characteristics

The research team designed the data collection process by reviewing the relevant literature and through their clinical experience. The data of the patients admitted to the surgery and gynecology departments of the hospital between August 2018 and January 2021 were included in an authentic electronic medical record management system, and the patients were screened and the electronic medical record and nursing document system data were retrieved. A total of 458 patients who met the inclusion criteria were enrolled, with their information checked by two professionals, including in terms of age, gender, body mass index (BMI),body surface arer (BSA), histology classification, complications, history of chemotherapy, family history of cancer, classification of perfusate, types of chemotherapy drugs, and laboratory inspection results. The patients were divided into the temperature increase group (axillary temperature ≥38°C) and the non-temperature-increase group (axillary temperature <38°C) according to the corresponding records of body temperature during HIPEC (**Table 1**).

**Table 1:** Demographics and clinical data of patients.

	All	Temperature rise group	Non-temperature rise group		
Characteristics	(n=458),%	(n=149),%	(n=309),%	*t/*χ^2^	*P value*
Age (year)				1.927	0.165
<60	243 (53.06)	86 (57.72)	157 (50.81)		
≥60	215 (46.94)	63 (42.28)	152 (49.19)		
Gender				17.253	<0.001
Male	176 (38.43)	37 (24.83)	139 (44.98)		
Female	282 (61.57)	112 (75.17)	170 (55.02)		
BMI (kg/m^2^)				2.81	0.422
<18.5	25 (5.46)	6 (4.03)	19 (6.15)		
18.5~23.9	184 (40.17)	55(36.91)	129 (41.75)		
24~28	182 (39.74)	62 (41.61)	120 (38.83)		
>28	67 (14.63)	26 (17.45)	41 (13.27)		
BSA(m^2^)	1.72±0.17	1.70±0.16	1.73±0.18	-1.731	0.084
Histology classification of tumor				33.961	<0.001
Appendix tumor	9 (1.96)	2 (1.34)	7 (2.27)		
Gastric cancer	196 (42.79)	37 (24.83)	159 (51.45)		
Colorectal cancer	55 (12.01)	21 (14.09)	34 (11)		
Pancreatic cancer	2 (0.44)	0 (0)	2 (0.65)		
Gynecological malignancies	183 (39.96)	84 (56.38)	99(32.04)		
Peritoneal malignant tumor/mesothelioma	13 (2.84)	5 (3.36)	8 (2.59)		
Complications				2.351	0.537
Hypertension	140 (30.57)	48 (32.21)	92 (29.77)		
Diabetes	54 (11.79)	23 (15.44)	31 (10.03)		
Cardiovascular and cerebrovascular diseases	57 (12.45)	19 (12.75)	38 (12.3)		
Liver diseases	12 (2.62)	6 (4.03)	6 (1.94)		
Perfusate				0.008	0.928
Glucose	19 (4.15)	6 (4.03)	13 (4.21)		
Normal saline	439 (95.85)	143 (95.97)	296 (95.79)		
Type of chemotherapeutic drug				48.536	<0.001
Paclitaxel	168 (36.68)	19 (12.75)	149 (48.22)		
Lobaplatin	200 (43.67)	86 (57.72)	114 (36.89)		
Cisplatin	46 (10.04)	19 (12.75)	27 (8.74)		
Fluorouracil	35 (7.64)	15 (10.07)	20 (6.47)		
History of chemotherapy				0.357	0.55
Yes	94 (20.52)	33 (22.15)	61 (19.74)		
No	364 (79.48)	116 (77.85)	248 (80.26)		
Family history of cancer				1.732	0.188
Yes	116 (25.33)	32 (21.48)	84 (27.18)		
No	342 (74.67)	117 (78.52)	225 (72.82)		
Preoperative PAlb (mg/L)	205.62±61.9	201.30±63.23	207.72±61.16	-1.041	0.298
Preoperative Alb (g/L)	40.45±4.91	40.52±3.97	40.41±5.31	0.248	0.804
Postoperative WBC (*10^9^/L)	10.50±3.99	10.98±3.67	10.27±4.12	1.789	0.074
NEUT% (%)	84.68±6.56	85.57±5.38	84.25±7.01	2.221	0.027
Postoperative RBC (*10^12^/L)	3.86±0.56	3.83±0.56	3.87±0.56	-0.716	0.474
Postoperative Hb (g/L)	111.76±18.9	109.62±16.18	112.78±18.99	-1.848	0.065
Postoperative PAlb (mg/L)	134.67±44.68	126.88±45.98	138.43±43.54	-2.611	0.009
Postoperative Alb (g/L)	29.28±4.39	28.79±4.15	29.57±4.48	-1.787	0.075

BMI, Body Mass Index (kg/m^2^).

BSA, Body Surface Area ( $\sqrt{\text{height}(\text{cm})*\text{weight}(\text{kg})}$ /60).

PA1b, Prealbumin.

A1b, Albumin.

WBC, White Blood Cell Count.

NEUT%, Neutrophil Percentage.

RBC, Red Blood Cell Count.

Hb, Hemoglobin.

### 3.2 Incidence of temperature increase following hyperthermic intraperitoneal chemotherapy

Around one-third of the patients (n = 149/458, 32.5%) suffered from a temperature increase, with an axillary temperature of not below 38°C. According to the results, the patients were characterized in terms of female gender, gynecological malignancies, intraperitoneal infusion of lobaplatin, increased postoperative neutrophil percentage (NEUT%) and a sharp drop in postoperative prealbumin (PAlb) (*P*< 0.05), while complications, perfusate selection, history of chemotherapy, and family history of tumors were deemed to be irrelevant factors (*P* > 0.05). Eighty patients were subjected to peripheral blood microbial culture, among which 20 patients (n = 20/80, 25.0%) returned a positive result, and 39 patients (39/458, 8.5%) high fever at an axillary temperature of >39°C.

### 3.3 Risk factors of temperature increase following hyperthermic intraperitoneal chemotherapy

According to the results, the differences in age, BMI, BSA, and temperature increase following HIPEC were not statistically significant among the patients (*P*> 0.05). The patients experiencing a temperature increase were comparatively younger (median [range] 57 [25–78]: 59 [20–85], *P* = 0.54). The difference between the patients with and without gynecological malignancies was statistically significant (*P*< 0.001). In terms of type of chemotherapy drug, 86 cases (86/149, 57.7%) administered with lobaplatin recorded a temperature increase, which was statistically different from those who did not use lobaplatin (*P*< 0.001). Meanwhile, only 19 (19/149, 12.8%) patients using paclitaxel had a body temperature of 38°C and above, and the difference was statistically significant (*P*< 0.001) (**Table 2**).

**Table 2:** Data of patient classification.

	All	Temperature rise group	Non-temperature rise group		
Characteristics	(n=458),%	(n=149),%	(n=309),%	*t/*χ^2^	*P value*
BMI	24.16±3.66	24.69±3.63	24.28±3.67	1.124	0.262
Age	57.1±11.5	56.6±10.9	57.3±11.7	-0.613	0.54
Lobaplatin				17.723	<0.001
Yes	200 (43.67)	86 (57.72)	114 (36.89)		
No	258 (56.33)	63 (42.28)	195 (63.11)		
Paclitaxel				54.448	<0.001
Yes	168 (36.68)	19 (12.75)	149 (48.22)		
No	290 (63.32)	130 (87.25)	160 (51.78)		
Gynecological malignancies				24.818	<0.001
Yes	183 (39.96)	84 (56.38)	99 (32.04)		
No	275 (60.04)	65 (43.62)	210 (67.96)		

BMI, Body Mass Index.

### 3.4 Logistic regression analysis for temperature increase following hyperthermic intraperitoneal chemotherapy

A logistic regression equation was created, with the occurrence of a temperature increase following CRS-HIPEC adopted as the dependent variable, and age, gender, gynecological malignancies, use of paclitaxel/lobaplatin as the chemotherapy drug, BSA, postoperative white blood cell count (WBC), NEUT%, and postoperative PAlb adopted as the independent variables. Age, BSA, WBC, NEUT%, and PAlb were inputted as original values, and the values of the other categorical data were assigned as follows: gender: male = 1, female = 0; gynecological malignancies: yes = 1, no = 0; use of paclitaxel: yes = 1, no = 0; and use of lobaplatin: yes = 1, no = 0.

The logistic regression analysis revealed that the type of chemotherapy drug was an independent risk factor for temperature increases following CRS-HIPEC (**Table 3**). Following data adjustment, the probability of a temperature increase in the patients receiving paclitaxel hyperthermic perfusion was 82.9% lower than that in those receiving lobaplatin, cis-platinum, and fluorouracil (odds ratio [OR] = 0.171, 95% confidence interval [CI]: 0.1–0.291; *P* < 0.001). In fact, the patients receiving lobaplatin were 5.9 times more likely to suffer from a temperature increase than those who were treated with paclitaxel (OR = 5.916, 95%CI: 3.401–10.29; *P* < 0.001). The relative risk of using lobaplatin was comparable to that of using cis-platinum (RR = 1.041, 95%CI: 0.712–1.522; *P* = 0.834). No significant difference was found in terms of the relative risk of using lobaplatin when compared with fluorouracil (RR = 1.003, 95%CI: 0.663–1.518; *P* = 0.987).

**Table 3:** Logistic regression analysis of CRS-HIPEC patients with temperature rise.

	*B value*	Standard error	wald*χ2 value*	Significance	Exp(B)	95% CI of EXP(B)	
						Lower limit	Upper limit
Age	-0.001	0.01	0.013	0.908	0.999	0.98	1.018
Gender	0.071	0.367	0.038	0.846	1.074	0.523	2.205
BSA	-0.174	0.731	0.057	0.812	0.840	0.201	3.519
Gynecological malignancies	-0.106	0.307	0.10	0.729	0.899	0.493	1.640
Paclitaxel	1.793	0.369	23.596	<0.001	6.006	2.914	12.38
Lobaplatin	0.181	0.254	0.509	0.476	1.198	0.729	1.970
WBC	-0.006	0.032	0.041	0.840	0.994	0.934	1.057
NEUT%	0.018	0.02	0.794	0.373	1.018	0.979	1.059
Postoperative PAlb	-0.003	0.003	1.224	0.269	0.997	0.992	1.002
Constant	-2.826	2.366	1.426	0.232	0.059		

WBC, White Blood Cell Count.

NEUT%, Neutrophil Percentage.

PA1b, Prealbumin.

Studies have revealed that CRS-HIPEC patients face certain nutritional risks. According to the present study results, the postoperative level of PAlb and Alb in the two groups was significantly lower than the preoperative level, with a statistical difference (*P*< 0.001) (**Table 4**).

**Table 4:** Comparison of nutritional indexes between the two groups.

Group	Preoperative PAlb (mg/L)	Postoperative PAlb (mg/L)	*t value*	*p value*	Preoperative Alb (g/L)	Postoperative Alb (g/L)	*t value*	*p value*
Temperature rise group (n=149),%	201.30±63.23	126.88±45.98	13.025	<0.001	40.52±3.97	28.79±4.15	24.931	<0.001
Non-temperature rise group (n=309),%	207.72±61.16	138.43±43.54	16.224	<0.001	40.41±5.31	29.57±4.48	27.427	<0.001

PA1b, Prealbumin.

A1b, Albumin.

## 4 Discussion

Different types of chemotherapy drug are independent risk factors for a temperature increase following HIPEC. In a multi-center HIPEC study carried out in China (8), the inflow temperature was set at 43°C and the perfusion duration was 90 min, and the patients’ recorded vital signs remained stabilized, with a sharp temperature increase and abdominal distension during HIPEC. After 30–90 min of perfusion, the patients’ heart rate, respiratory rate, and rectal temperature (as high as 39.7°C–40.2°C) were all above baseline level before returning to the baseline within 30 min following perfusion; however, the study did not discuss the effect on temperature increase by different chemotherapy drugs. A study by Guerra-Londono (9)demonstrated that the effect of chemotherapy drugs on temperature increase is significant (*P*< 0.001), with mitomycin found to be an independent prognostic factor for mild hyperpyrexia (≥38°C), and patients using cis-platinum 76.5% less likely to experience a temperature increase to ≥38°C than those using mitomycin (*P* = 0.036). Elsewhere, Ye (10) enrolled 59 CRS-HIPEC patients, among which 30 patients used cis-platinum for perfusion and as many as 64.4% suffered from a temperature increase following HIPEC.

In clinical practice, it is crucial to constantly summarize the risk factors of hyperthermia caused by different types of chemotherapy drugs during HIPEC to ensure that the medical staff can take preventive measures to prevent the adverse effects of metabolic and hemodynamic changes caused by hyperthermia. Consensus guidelines for perioperative management of CRS-HIPEC patients (11) suggest that maintaining a low body temperature is an important objective, one that can be realized by placing an ice bag under the patient’s armpit or at the head and neck arteries. When the core temperature reaches 39°C and above, lowering the temperature of the perfusate could be a practical method. Meanwhile, the nursing staff must closely observe and record any changes in the patient’s body temperature during HIPEC, and report these to the doctor immediately, such that appropriate measures can be taken when the body temperature reaches 38°C, ensuring that the patient’s body temperature does not fluctuate sharply.

Following CRS-HIPEC, a complicated surgery for patients with malignancies, while according to Gusani et al. (12), abdominal complications (abscesses, fistulas, and anastomotic leaks) are the most common grade III or IV complications. According to the criteria issued by the National Cancer Institute, the incidence of grade III or IV complications is 29.8%, and the incidence of complications combined reaches 56.5%. In the study by Cripe et al. (13), following CRS-HIPEC, the incidence of grade III/IV complications among patients with gynecological malignancies was 65.6%, with the most common complications anemia (40.6%), infection (15.6%), and pleural effusion (12.5%). abdominal infection is one of the most common complications during postoperative recovery. The attendant postoperative incidence rate is 12%–52%, a key reason why patients can face an unexpected re-operation. Bacterial translocation arising from HIPEC is a potential cause of postoperative infection complications (14).

The present study revealed that following surgery, the NEUT% in the temperature increase group was higher than that in the non-temperature-increase group (*P* = 0.027), indicating a higher risk of infection, which, in turn, is a risk factor for temperature increases following HIPEC. Multiple inflammatory mediators produced in complicated surgeries can induce systemic inflammatory responses, with the relative severity potentially affecting the clinical outcome. Following HIPEC, all the patients suffered from severe adverse reactions, with rapidly increased interleukin and C-reactive protein (CRP) (15). Interleukin-6 (IL-6) is both a pro-inflammatory and anti-inflammatory factor, playing a key role in immune response and cell proliferation and apoptosis. During HIPEC, the IL-6 in the plasma significantly increased from baseline to the beginning of the perfusion (*P*< 0.01), maintaining a high level for a long period up to day 7 post-surgery (16). As a commonly used biomarker in clinical practice, CRP is accurate, cheap, and easy to measure, presenting a measure for the intensity of inflammatory response and also involved in the activation of inflammatory cascade, thus potentially predicting postoperative complications pertaining to abdominal surgery.

Previous studies have found that patients with postoperative complications have a higher level of CRP, which is associated with a higher complication grade (17). Dazza (18) carried out a bacteriologic analysis of intraperitoneal flushing fluid (RLBA) obtained following CRS-HIPEC and found that 40% of the RLBA specimens returned a positive bacterial test result. The author concluded that a positive RLBA result is a predictive factor for postoperative complications of abdominal infection. Elsewhere, studies (19) have demonstrated that complications following CRS-HIPEC (infectious, cardiopulmonary, and thrombotic complications and gastrointestinal motility disorder) are associated with decreased overall survival and progression-free survival rates, with researchers finding that infectious complications are the main driving force behind such an association in all types of complications. Therefore, best practices and standardized prevention strategies must be adopted to minimize the incidence of such infectious complications following surgery. Selecting targeted, sensitive treatments with antibiotics, timely upgrading the antibiotics (14, 16, 18, 19), administering them as scheduled, and maintaining a stable, effective plasma concentration in the blood are key measures during the perioperative period in clinical practice.

Other factors may also affect the body temperature of HIPEC patients. Gabriel E (20) demonstrated the fact worth paying attention to in the future research, that after the HIPEC cycle, lavage with normal saline at room temperature could not only remove circulating chemotherapy drugs, and it would also be expected to decrease whole body temperature. Furthermore, this study did not investigate the effect on the patients’ temperature of different surgical methods: open versus minimally invasive surgery (MIS). Morton M (21) found that in women with advanced epithelial ovarian cancer (EOC), HIPEC with MIS at the time of interval debulking surgery (IDS) following NACT is feasible, compared to open the similar rates of R0 cytoreduction. Gabriel E (22) first reported video of a robot treating gastrointestinal malignancies CRS-HIPEC, the roboticassisted approach for CRS-HIPEC is a feasible option for the highly select patient. With many more centers performing MIS surgeries, surgical approach may impact temperature regulation.

Nutritional intake during the CRS-HIPEC perioperative period affects the postoperative outcome. A study by Reece (23) demonstrated that 34 (33%) of the 102 patients enrolled were classified as malnourished (subjective global assessment = B/C). These malnourished patients recorded significantly increased body loss (15%: 74%; *P*< 0.001) and the presence of clinical symptoms (18%: 47%; *P* = 0.002). The malnutrition itself was significantly associated with postoperative infection complications and increased LOS, increasing by an average of 7.65 days for each worsening grade of malnutrition. The main risk factors for non-home-discharge/LOS following CRS-HIPEC are advanced age, hypoalbuminemia, and multivisceral resection. Furthermore, malnutrition is also a relevant factor for patient re-admission (24, 25).

According to the present study results, the postoperative level of PAlb and Alb in the two groups was significantly lower than the preoperative level (*P*< 0.001). Therefore, early postoperative nutritional intervention is essential, with enteral nutrition the first choice for nutritional supplementation since it helps maintain the intestinal function and reduce any bacterial translocation. Malnourished patients or those estimated to be at risk of malnutrition for longer than three days must be administered parenteral nutrition as early as possible (26).

The enhanced recovery after surgery (ERAS) program has been widely used among perioperative patients to promote their postoperative recovery. In fact, the ERAS program is also feasible for CRS-HIPEC patients, and is not associated with major complications or an increase in re-admission rates (27). The guidelines for ERAS perioperative nursing of CRS with or without HIPEC provide specific measures, as well as guidance and suggestions for clinical practice (28, 29).

This study has certain limitations. First, it was a single-center, retrospective study, involving multiple cancer types and different departments. As such, the surgical scopes were heterogeneous, the operators performing the perfusion were from different teams, and the specimens were tested at different time points following the surgery, which may have affected the accuracy and reliability of the data collected. Furthermore, the study lacked a prospective predictive design for the influence of temperature increases following CRS-HIPEC. Further clinical studies are needed to verify the applicability and universality of the findings among the same species of disease, in addition to exploring different risk factors for temperature increases following CRS-HIPEC.

## 5 Conclusion

The influence of different types of chemotherapy drugs is an independent risk factor for a temperature increase following CRS-HIPEC. Understanding the properties and indications of different drugs and the clinical test results of HIPEC patients can help medical workers better identify the risks pertaining to temperature increases, allowing them to take preventive measures in a timely manner in view of maintaining the patient’s body temperature as stable during HIPEC. In addition, in-depth clinical research must be carried out to construct risk-prediction models for temperature increases following CRS-HIPEC.

## Data availability statement

The original contributions presented in the study are included in the article/supplementary material. Further inquiries can be directed to the corresponding author.

## Ethics statement

The studies involving human participants were reviewed and approved by Ethics Committee of The Fourth Hospital of Hebei Medical University. The patients/participants provided their written informed consent to participate in this study.

## Author contributions

J-YM, SK and H-XK conceived the idea and conceptualized the study. Y-YS, H-XK, B-JF, X-BF, X-SW and Y-YL collected the data. Y-YS, B-JF, X-BF, X-SW, Y-YL, J-YM, SK and H-XK analyzed the data. H-XK drafted the manuscript, then J-YM, SK and H-XK reviewed the manuscript. All authors read and approved the final draft.
